# Protein, Essential Amino Acid, and Fatty Acid Composition of Five Target Fishery Species of Central Mediterranean Sea

**DOI:** 10.3390/ani14152158

**Published:** 2024-07-24

**Authors:** Anna Traina, Enza Maria Quinci, Nadia Sabatino, Marianna Del Core, Antonio Bellante, Gioacchino Bono, Marta Giuga, Giuseppe Avellone, Mario Sprovieri, Fabio D’Agostino

**Affiliations:** 1 National Research Council of Italy, Institute of Anthropic Impacts and Sustainability in Marine Environment (CNR-IAS), 90149 Palermo, Italy; anna.traina@cnr.it (A.T.);; 2 National Research Council of Italy, Institute of Anthropic Impacts and Sustainability in Marine Environment (CNR-IAS), Torretta Granitola-Campobello di Mazara, 91021 Trapani, Italyfabio.dagostino@cnr.it (F.D.); 3 National Research Council of Italy, Institute for Biological Resources and Marine Biotechnology (CNR-IRBIM), Mazara Del Vallo, 91026 Trapani, Italy; 4 Department of Biological, Chemical and Pharmaceutical Sciences and Technologies (STEBICEF), University of Palermo, Via Archirafi 32, 90123 Palermo, Italy; 5 National Research Council of Italy, Institute of Anthropic Impacts and Sustainability in Marine Environment (CNR-IAS), 16149 Genova, Italy; 6 National Research Council of Italy, Institute of Marine Science (CNR-ISMAR), 30122 Venezia, Italy

**Keywords:** seafood quality, EPA, DHA, nutritional indexes, southern Italy, NMDS model

## Abstract

**Simple Summary:**

Fishery products are an important part of the Mediterranean diet and constitute a high source of nutrients for consumers, contributing to the prevention of several health diseases. This work analysed the protein, amino acids, and fatty acids of Mediterranean target species (four fish species and one crustacean species) which are mostly consumed in Sicily. The nutritional composition of the selected species together with the evaluation of nutrient indices showed the high quality of the sampled fish. The excellent profile of the fatty acid of “blue fish” species highlights the opportunity for local people to benefit from a highly nutritional product originating from local and sustainable fisheries and with an affordable commercial value.

**Abstract:**

The protein, essential amino acid, and fatty acid composition of European pilchard (*Sardina pilchardus*), European hake (*Merluccius merluccius*), surmullet (*Mullus surmuletus*), red mullet (*Mullus barbatus*), and deep water rose shrimp (*Parapenaeus longirostris*) from the central Mediterranean Sea were investigated. All the species showed an essential amino acid content of about 50% of total amino acids, while the protein and total fatty acids content varied from 19.9 to 24.8% and from 1.4 to 5.1%, respectively. The fatty acid profile mainly followed the order SFA (39.1–52.6%) > PUFA (21.0–39.3%) > MUFA (15.6–24.3%). Palmitic and stearic acids were predominant among saturated fatty acids (38–52% and 21–25%, respectively), while palmitoleic and oleic acids were the most represented of the total monounsaturated acids (10–21% and 55–68%, respectively). All the species, as expected, showed a more significant proportion of n-3 PUFA (EPA + DHA) of about 81–93% of the total PUFA, with the highest values was found in European pilchard. Also, several fat quality index values, such as n-6/n-3 ratio, PUFA/SFA, the index of atherogenicity (IA), the index of thrombogenicity (IT), the hypocholesterolemic/hypercholesterolemic ratio (HH), and fish lipid quality/flesh lipid quality (FLQ) were calculated to assess the nutritional quality. All the obtained results, along with the fat quality indexes, indicated the excellent nutritional values of the selected species.

## 1. Introduction

Fishery products are largely considered healthy foods, representing the primary source of omega-3 polyunsaturated fatty acids (n-3 PUFAs) and contributing to the intake of numerous nutrients [1,2,3]. This awareness/perception is deeply rooted in the central Mediterranean communities, where fishery products (bony fish, crustaceans, cephalopods, and elasmobranchs) are a regular part of the well-known “Mediterranean diet” [4]. The protein and lipid contents of the edible part of fishery products make them nutritionally valuable and a valid substitute for other meals based on animal sources (such as red meat, pork, poultry, and seafood products from intensive aquaculture).

Several research studies have highlighted the importance of the effect of fish proteins in maintaining significant body functions and preventing health diseases related to metabolic syndromes, inflammation, or insulin resistance [5,6,7,8]. Fish proteins are also rich in essential amino acids (EAAs) such as Valine, Leucine, Isoleucine, Threonine, Phenylalanine, Lysine, and Tryptophan [9,10]. EAA intake is very important since they have a high biological value, regulating key metabolic pathways and being associated with the prevention and treatment of several organism’s diseases and dysfunctions, such as obesity and diabetes, infertility, growth limitation, and intestinal, neurological, and renal dysfunctions [11,12,13].

PUFAs of the n-3 group, represented by eicosapentaenoic acid (EPA) and docosahexaenoic acid (DHA), are well known to exert several beneficial effects on human health, preventing cardiovascular, autoimmune, and neural diseases [14,15,16]. Thus, seafood consumption has been demonstrated to exert health benefits in humans despite the potential risks due to contaminant bioaccumulation, especially in fish tissues [17,18,19], and/or the emerging threat of marine micro- and nanoplastic pollution [20].

Nevertheless, the higher consumption of processed food and vegetable oils in the modern Western diet results in a higher intake of n-6 PUFA [21,22]. This higher intake of n-6 PUFA is known to be responsible for many health diseases [23,24], such as an increased incidence of neurodegenerative and neurological disorders [25]. Therefore, increasing n-3 and reducing n-6 consumption to reduce the n-6/n-3 ratio between 5:1 and 10:1 is recommended for beneficial effects regarding human health [24,26,27]. The fatty acid composition and content of marine fish can vary depending on various factors, such as diet, age, size, reproductive cycle, and environmental conditions [27]. Thus, the knowledge of fatty acid content in different species, together with the evaluation of several nutritional indices, could be useful for consumers to identify highly healthy species, enabling them to make the best choices for their health.

Some low-valued fishery products, as well as by-catches of massive fisheries [28,29], have the advantage of being, at the same time, highly nutritive food and easily accessible at low or no cost for people with low or zero income [11]. This may be a strong tool to reduce worldwide malnutrition [30], providing at least 20% of protein intake for a third of the world’s population that lives under poverty [31]. Under this framework and based on landed production data provided by the DCF (Data Collection Framework; https://datacollection.jrc.ec.europa.eu, accessed on 26 June 2020), we determine the nutritional composition (i.e., total protein and fatty acids) of five target species of the Italian commercial fishery from different markets in order to evaluate the advantages for human health and the differences between species coming from different sampling areas, consequently determining the most suitable for human consumption.

## 2. Materials and Methods

### 2.1. Samples Strategy and Collection

**Figure 1:**
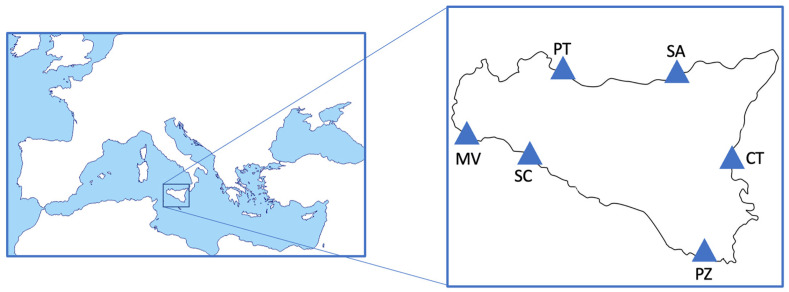
Chart of the Mediterranean Sea indicating the Sicilian fish markets.

Five target species have been selected based on their total landing weight and/or related economic value. Specifically, one small pelagic species such as European pilchard (*Sardina pilchardus*), three demersal bony fish species such as European hake (*Merluccius Merluccius*), surmullet (*Mullus surmuletus*), and red mullet (*Mullus barbatus*), and one crustacean species such as deep water rose shrimp (*Parapenaeus longirostris*) were directly collected with the support of professional local fishermen from some major *Sicilian* fish landing ports (southern Italy) (Figure 1). Six ports were identified as follows: Porticello (PT) and S. Agata (SA) on the northern coast (the southern Tyrrhenian Sea), Catania (CT) on the east coast (Ionian Sea), and Mazara del Vallo (MV), Sciacca (SC), and Pozzallo (PZ) on the southern coast (Strait of Sicily Sea) (Figure 1), with the first aim of evaluate potential differences in the nutritional values of the selected species from different areas. After their collection, samples were packaged in a sealed polyethylene box and quickly frozen and stored at −20 °C. Once in the laboratory, biometric parameters, such as total length (cm) for fish and carapace length (cm) for crustaceans and weight (g), were measured for each specimen (Table 1). Specimens were dissected after thawing by means of stainless-steel scissors in order to avoid contamination. The edible part (muscle), cleaned by skin or carapace, was collected, and different pools (each composed between 1 and 30 organisms, depending on the species) were obtained. Finally, samples were homogenised for a few seconds with a knife mill (Retsch-grindomix GM 200) and set for biochemical analysis.

### 2.2. Protein and Amino Acids Content

Considering that the protein content of fish flesh, unlike the fat content, is very constant, independent of seasonal variations and feeding and reproductive cycles, and that only small differences between species are present, protein measurements were performed on a singular pool of each species from different areas consisting of homogenised tissue [32]. The protein determination was calculated by nitrogen values (%) determined by a Thermo Electron Flash EA 1112. The fresh samples (ca. 0.5 mg) were packed into tin capsules and run against an internal standard (acetanilide with C = 71.09%, N = 10.36%). The crude protein was determined according to the Association of Official Analytical Chemists by converting the nitrogen content (N × 6.25) [33]. Based on six analyses of the homogenised fish tissue, the repeatability test was carried out and the value was below 2%. The amino acid composition in fresh tissue was determined by ethyl chloroformate (ECF) derivatisation reagent [34] and using a GC/FID instrument (GC TRACE 1310 and Triplus 100LS Autosampler, purchased by Thermo Fisher Scientific, San Jose, CA, USA). Standard solutions were prepared to calibrate the instrument method and perform the calibration curves using the standard mixture 79248 (by Sigma Aldrich, Milan, Italy) composed of 20 amino acids. This standard was appropriately diluted with distilled water at several levels for each amino acid at 0.025 mM, 0.05 mM, 0.125 mM, 0.25 mM, and 0.5 mM, and the samples were treated as described below. About 10 mg of the freeze-dried samples were hydrolysed at 110 °C for 24 h with 200 µL of 6 M HCl in an autoclavable vial. After the vial was cooled to room temperature and the solution was dried under a gentle N_2_ current flow, the residues were re-dissolved in 150 µL of distilled water and 150 µL of chloroform. After manual agitation using a glass capillary directly in the vial and waiting for the formation of two phases, the chloroform phase was discarded, and 100 µL of the water solution was transferred into a second micro-vial to derivatise the solubilised amino acids. This 100 µL of solution of each sample or 100 µL of each level of the amino acid standard solution was treated as follows: (i) mixed with 50 μL of a 4:1 solution of ethanol and pyridine; (ii) added 10 μL of ECF which was agitated slowly due to the released CO_2_ gas, causing effervescence and consequently losing part of the sample; (iii) added 50 μL of a 1% ethyl chloroformate solution in chloroform; and (iv) 50 μL of the saturated NaHCO_3_ solution was added to neutralise the solution. At the end of this procedure, each sample consisted of an aqueous top layer and a chloroform bottom layer. The lower organic layer was anhydrified by adding anhydrous sodium sulphate and was transferred to a fresh vial for the following GC/FID analysis. The amino acid was determined with a GC/FID Trace 1310 instrument (by Thermo Fisher Scientific) using a capillary column TR-1701 (Thermo Fisher Scientific) of 30 m × 0.25 mm × 0.25 µm and helium (He) carrier gas at a flow rate of 1.0 mL·min^−1^; the PTV injector was set in split mode (10:1) at 280 °C; and the FID detector temperature was 300 °C. The oven thermal gradient programme was set as follows: starting at 100 °C and held for 2 min, then ramped at 10 °C·min^−1^ to 270 °C, followed by a ramp, and held for 10 min. As for protein determination, the repeatability test for amino acid measurement was run, and it was between 8 and 12%.

### 2.3. Fatty Acids Profile Determination 

The fatty acid (FA) profile was determined as fatty acid methyl esters (FAMEs) based on the lipid extraction reported by the Bligh and Dyer method [35]. The fresh fish tissue of each pool per site was treated following these steps: about 1 g of the thawed sample was weighed directly in a glass vial; 10 mL of a chloroform/methanol solution (1:1 *v*/*v*) was pooled, capped, and placed in an ultrasound bath for 30 min; and in the same vial, another aliquot of 5 mL of chloroform was added and placed another time in an ultrasound bath. The solution containing the fish tissue fragments was filtered using a fast filter paper and pooled in a second vial. The residual was washed with another 5 mL of chloroform, and the paper filter was squeezed to quantitatively separate the lipid extracted from the tissue fragment and pool it in the previous second vial. After that, a chloroform clean-up procedure was followed, with about 5 mL of 1% saline water to remove methanol, polar substances, and co-extracted water-soluble impurities. After two layers had been formed, the upper water solution was discarded, and the chloroform solution containing lipid extract was anhydrified by adding a spoonful (about 0.5 g) of anhydrous sodium sulphate (Na_2_SO_4_) and was then dried using a vacuum multivapor. Finally, 1 mL of hexane was added to re-dissolve the lipid extracted using a vortex mixer for 30 s. The FAMEs were obtained by a trans-esterification reaction: 0.2 mL of 2 M alcoholic potassium hydroxide (KOH) was added to the hexane solution directly into the previous vial, agitated by vortex for 30 s, and allowed at least 30 min to complete the reaction. The same vial was placed in the TriPlus autosampler of the GC/FID Trace 1310 instrument (Thermo Fisher Scientific). The fatty acid was determined with a GC/FID using a capillary column TR-FAME (Thermo Fisher Scientific) of 100 m × 0.25 mm × 0.1 µm and helium (He) carrier gas at a flow rate of 1.5 mL min^−1^; the PTV injector was set in split mode (20:1) at 280 °C; and the FID detector temperature was 300 °C. The oven thermal gradient programme was set as follows: starting at 100 °C (held for 2 min), then ramped at 2 °C min^−1^ to 235 °C (held for 5 min), followed by a ramp to 260 °C ramped at 40 °C (held for 5). The determination of FAMEs in the samples was identified by comparing the retention time for each fatty acid with the retention time measured using a Supelco MIX37 Component FAMEs mix standard (Supelco, Bellefonte, PA, USA). The instrument’s calibration to quantify each fatty acid was developed using four levels of Mix37, as mentioned above, appropriately diluted with hexane.

The sum of each fatty acid determined the total FAs, and the profile was expressed as a percentage with respect to the total FAs.

### 2.4. Nutritional Quality Indexes

Different nutritional indexes based on fatty acid compounds were used to evaluate the lipid quality [36] of the selected species. The n-6/n-3 ratio was calculated as Σ n-6 PUFA/Σ n-3 PUFA; PUFA/SFA refers to the fraction of PUFAs over SFAs; the index of atherogenicity (IA) and thrombogenicity (IT), the Hypocholesterolemic/hypercholesterolemic ratio (HH), and the fish lipid quality (FLQ) were calculated as follows [37,38,39]:
IA: [C12:0 + (4 × C14:0) + C16:0]/ΣUFAIT: (C14:0 + C16:0 + C18:0)/[(0.5 × ΣMUFA) + (0.5 × Σn-6 PUFA) + (3 × Σn-3 PUFA) + (n-3/n-6)]HH: (*cis*-C18:1 + ΣPUFA)/(C12:0 + C14:0 + C16:0)FLQ: 100 × (C22:6 n-3 + C20:5 n-3)/ΣFA
where MUFA stands for monounsaturated fatty acids; PUFA stands for polyunsaturated fatty acids; UFA stands for unsaturated fatty acids; and FA stands for fatty acids

### 2.5. Statistical Analysis 

Significant differences in the total protein and essential amino acid percentages were tested among species using Kruskal–Wallis tests. A post hoc Dunn test was performed in the case of statistically significant results to determine which species differed from each other.

Nonmetric multidimensional scaling analysis (NMDS), based on the Euclidean distance matrix, was applied to categories of fatty acids (SFA, MUFA, PUFA n-3, PUFA n-6) to assess if the composition of the nutritional parameters were linked to particular species or fish markets. The final result was the transformation of the original multivariate dataset into a space with a reduced number of dimensions, visualised in an ordination diagram with two axes. The performance of the final model was measured through the “stress value” [40], which indicates the difference between the dissimilarities in the reduced dimension when compared to the complete multidimensional space. The correlation between the variables and axes was represented in the ordination plot by means of arrows with a length proportional to its strength. The closeness between points indicated how similar the fish individuals were among them based on fatty acid composition.

The calculated indices were plotted through boxplots by species and fish markets to assess possible different nutritional qualities. The significance of these differences was performed using Kruskal–Wallis tests. All statistical analyses were implemented using the statistical software R 4.1.0 [41].

## 3. Results and Discussion

### 3.1. Protein Content and Essential Amino Acids

The average percentage of total proteins ranged from 19.9 to 24.8% of wet weight (w.w.) registered in European hake and European pilchard, respectively (Table 2). No statistically significant difference in amino acid percentage was found among species (KW = 4.81; *p*-value > 0.05), while the percentage of total proteins differed significantly between European hake and European pilchard (Dunn test = −3.36; *p*-value < 0.05) and between deep water rose shrimp and European pilchard (Dunn-test = −3.26; *p*-value < 0.05). The Daily Recommended Dietary Allowance (RDA) of proteins indicated for an adult (60 kg) by the World Health Organisation (WHO) is 0.8 g protein/kg body weight [42]. Considering a 150 g portion of the selected species, the contribution to the recommended daily protein amount is 63% for European hake and deep water rose shrimp, 72% for red mullet and surmullet, and 78% for European pilchard.

High-quality proteins are readily digestible and contain the dietary EAAs in amounts that correspond to human needs [42]. We reported the content of EAAs measured in the selected species because it is the main determinant of the protein nutritional quality [43]. The average fraction of EAAs in analysed seafood samples constitutes from 48 to 52% of the total amino acidic content. Among the analysed EAAs, leucine was the predominant fraction (2.2–3.2% w.w.). Our results indicate that all the selected species constitute a rich source of healthy protein and essential amino acids which is able to reach the recommended daily dose.

### 3.2. Fatty Acids Profile

The total fatty acids (TFAs) were expressed as a percentage of wet weight (% w.w.) and was calculated as the sum of singular FAs. Individual FA values (% of TFAs) measured in selected species are shown in the Supplementary Materials (Tables S1 and S2), while the content of SFA, MUFA, PUFA, EPA, and DHA are shown in Table 3. The TFA content differs among the marine species analysed, ranging from 1.44 to 5.05%, as registered in European hake and European pilchard, respectively. According to Ackman (1990) [44], fish can be grouped into four categories depending on their fat content as follows: lean fish (<2%), low fat (2–4%), medium fat (4–8%), and high fat (>8%). Among the analysed organisms, surmullet, red mullet, European hake, and deep water rose shrimp were considered to be low-fat species, while European pilchard showed a lipid content comparable to a medium-fat fish (5.05 ± 2.52 w.t.) (Table 3).

Palmitic (C16:0) and stearic acids (C18:0) were the predominant saturated fatty acids (SFA) (38–52% and 21–25%, respectively) present in all species, according to results from several studies focused on the same species [45,46,47,48,49] and, in general, on marine fish species [50,51]. Among the selected species, European pilchard contained the smallest amount of both palmitic (C16:0) and stearic (C18:0) acids of TFAME, (17.61% and 7.90%, respectively), while the highest proportion of C16:0 (24.80%) and C18:0 (12.40%) was found in surmullet and in red mullet, respectively (Tables S1 and S2).

**Figure 2:**
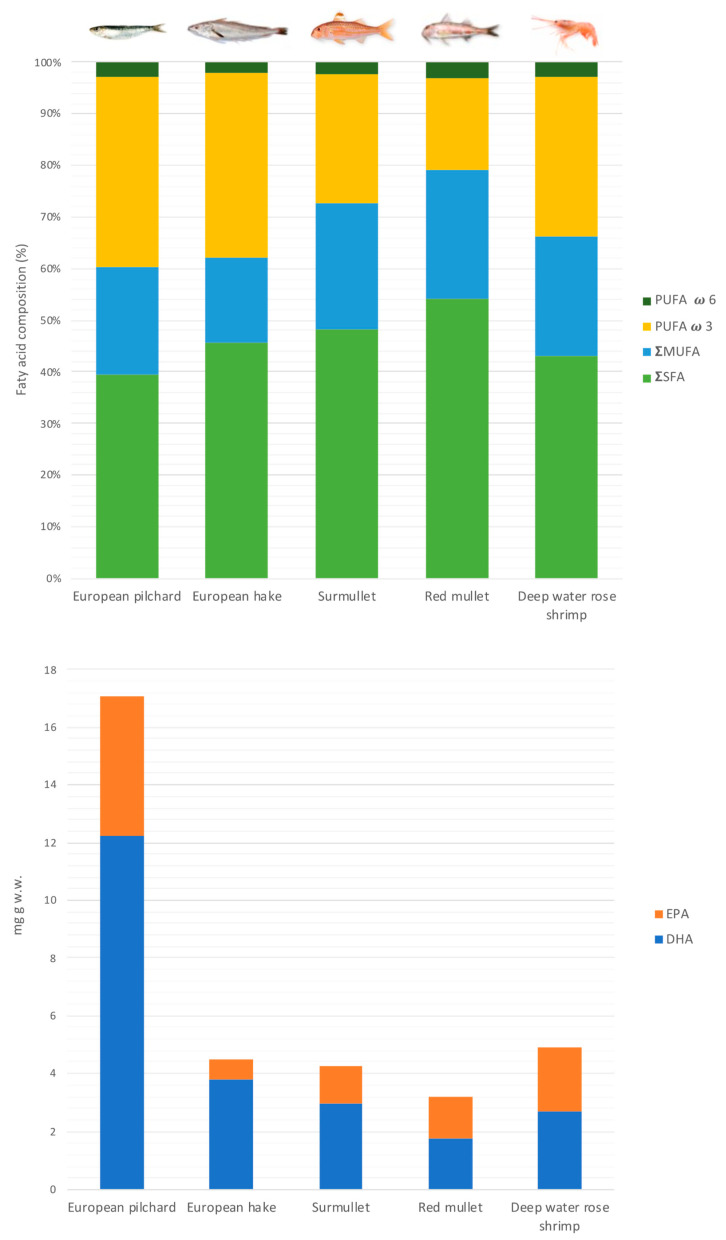
(**bottom**) EPA and DHA content (mg g w.w.) and (**top**) fatty acid proportion (%) in the analysed species.

Among the monounsaturated fatty acids, the palmitoleic (C16:1 n-7) and oleic (18:1 n-9) acids were the most represented, accounting for 10–21% and 55–68% of total MUFA, respectively, as reported from various studies on marine species [49,50,52] (Tables S1 and S2 and Table 3). The total PUFA varied from 21.0% to 39.3%, as registered in red mullet and European pilchard, respectively. Eicosapentaenoic (20:5 n-3, EPA) and docosahexaenoic (22:6 n-3, DHA) were the most abundant, accounting for 63–85% of the total PUFA. Generally, DHA assumed higher values than EPA in all the species analysed (Table 3). The highest value of EPA was detected in deep water rose shrimp (11.59% of FA) and the lowest in European hake (4.91%), whereas the highest content of DHA was found in European hake (26.28%) and the lowest in red mullet (7.57%). These results are in agreement with other works focused on the same species [49,53,54]. The European Food Safety Authority (EFSA) suggests a daily intake of EPA and DHA consumption between 0.25 and 0.5 g in order to protect against the risk of cardiovascular diseases [55]. The highest EPA and DHA content (mg g w.w.) was found in European pilchard, followed by deep water rose shrimp, European hake, surmullet, and red mullet (Figure 2, bottom). Based on data in Table 3, and considering a fish portion of 150 g, the EPA + DHA values were 0.48, 0.64, 0.67, 0.73, and 2.56 g per portion of red mullet, surmullet, European hake, deep water rose shrimp, and European pilchard, respectively. Figure 2, top, shows the proportion of the total fatty acid composition in the analysed species following the order SFA (39.1–52.6%) > PUFA (21–39.31%) > MUFA (15.63–24.34%). Only the red mullet showed a content of MUFA slightly higher than PUFA (24.34 and 21%, respectively), as found by Prato and Biandolino [46].

Both n-6 and n-3 PUFA play crucial functions in regulating several biochemical and physiological processes with opposite effects [56]. Since they are not synthesised by the organism, they must be assumed from the diet. Thus, an unbalanced fatty acid intake can result in the occurrence of several diseases [24,57].

The n-3 PUFA is a vital structural constituent of the phospholipid cell membranes participating in cell regulation and playing a crucial role in preventing many disorders such as cardiovascular, atherosclerosis, heart attack, depression, cancer, neural, and autoimmune diseases [13,15,24,58,59,60,61,62,63,64,65]. On the contrary, previous studies found that a higher intake of n-6 PUFA is often associated with adverse effects on human health, such as inflammatory processes, cancer, autoimmune, and cardiovascular disorders [66,67,68,69]. All the analysed species contain a higher proportion of n-3 PUFA with respect to n-6 PUFA, representing 81–93% of the total PUFA. The highest content of n-3 PUFA was registered in European pilchard (36.27%) and the lowest in red mullet (17.37%).

**Figure 3:**
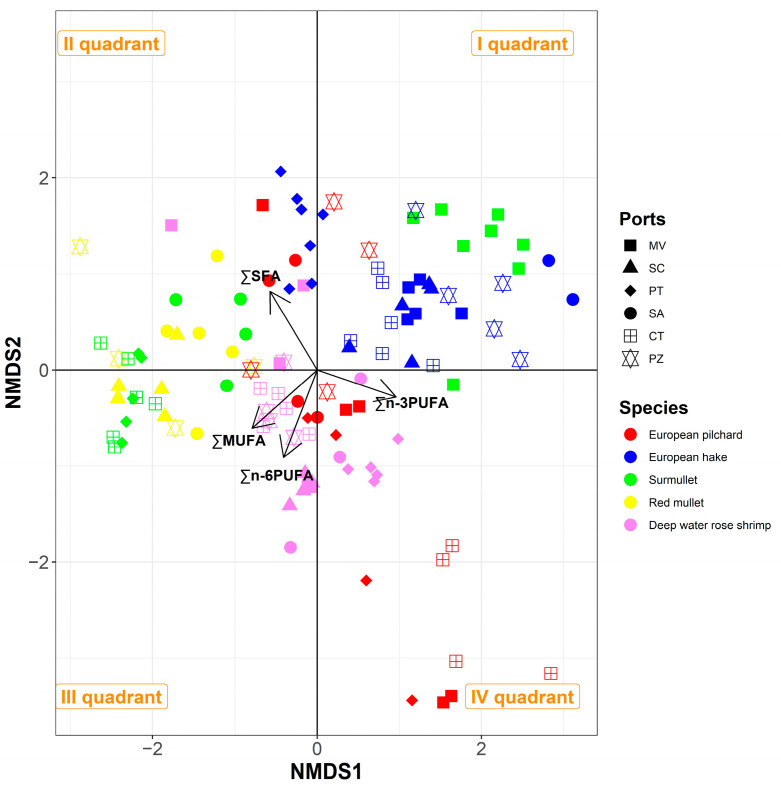
Ordination plot generated by NMDS model applied to fatty acid content in specimens from different fish markets. The points are represented with different colours and shapes, corresponding to a specific fish market and species, respectively.

The statistical approach using the NMDS model allowed for the identification of differences in fatty acid composition among the species and sampling areas. The stress value was smaller than 10%, indicating a good representation of the original data. All variables were significantly correlated with the axes. Scores from NMDS along the two first dimensions are graphically represented in Figure 3. The samples are clearly clustered in the four quadrants of the plot. One cluster (I quadrant) is composed of European hake from all sites except PT, surmullet sampled in MV, and European pilchard from PZ. This cluster does not have a predominance of a particular fraction of fatty acids, but it is characterised by low MUFA and n-6 PUFA values. The second cluster (II quadrant), characterised by high SFA values, is composed of European hake from PT and surmullet and red mullet from SA. The third cluster (III quadrant), with high MUFA and n-6 PUFA values, is composed of red mullet from SC and deep water rose shrimp from CT and SC. The fourth cluster (IV quadrant) includes European pilchard individuals collected in CT, PT, and MV and deep water rose shrimp from PT and SA. This cluster is dominated by high levels of n-3 PUFA and low values of SFA, MUFA, and n-6 PUFA. Moreover, some samples are located along the negative side of the horizontal axis because they are dominated by SFA, MUFA, and n-6 PUFA, with low n-3 PUFA values. This group includes surmullet from CT and PT and red mullet from SC and PZ.

Some species such as deep water rose shrimp, red mullet, and European hake are clustered together within the plot, indicating similar characteristics regardless of their origin. Differently, the surmullet and European pilchard are spread across multiple quadrants. Specifically, the group formed by surmullet from MV is located in a different quadrant (I quadrant) when compared to specimens from the other areas, therefore indicating distinct characteristics. Similarly, the group consisting of the European pilchard from CT diverges from the specimens of the same species from different areas. These results are probably due to both the intrinsic characteristics of the species and the availability of food in the area. Indeed, although marine fish could synthesise PUFAs (EPA and DHA) through characteristic enzymatic pathways, this enzymatic activity is low, and their content of PUFA appears to be closely related to diet [70,71]. The primary production of PUFA can occur both in pelagic and benthic algae [72,73], and microalgae constitute an important source of these marine bioactive materials [74]. Furthermore, oceanographic features play a key role in the distribution of primary producers, as well as in habitat characterisation and the availability of infauna, affecting the diet of marine species. The variability of the nutritional composition of fish depends on several biological parameters, such as different age, size, and the maturation status of the fish, and by different habitat characteristics, such as water temperature, food availability, and fishing impact [47,51,75,76,77,78,79,80,81]. The combination of these features easily explains the different fatty acid distributions in specimens of the same species coming from different fishing areas of the Mediterranean Sea.

### 3.3. Nutritional Quality Indices

Several fat quality indexes (Table 4), such as the n-6/n-3 ratio, PUFA/SFA, the index of atherogenicity (IA), the index of thrombogenicity (IT), the hypocholesterolemic/hypercholesterolemic ratio (HH), and fish lipid quality/flesh lipid quality (FLQ), can be useful to assess the contribution of FAME towards human health [37,38,82,83,84]. In this study, we found a n6/n3 ratio between 0.06 in European hake and 0.19 in red mullet, thus being lower than the value of 4.0 recommended by the UK Department of Health [84]. This result indicates that the selected species contribute to a proper intake of these fatty acids (Table 4).

The PUFA/SFA ratio reflects the effects of PUFA and SFA on human health [82]. A minimum value ratio of 0.45 is recommended for one’s diet since a higher value may result in increased blood levels of cholesterol [84]. The PUFA/SFA ratio measured in the selected species was higher than the recommended value in all the species analysed except for red mullet (0.41), probably because of his lower PUFA content (21% of FAME) (Table 2 and Table 3). However, it should be kept in mind that these indices may underestimate the quality of fatty acids, providing simplistic guidance. Generally, the ratio of PUFA/SFA or n-6/n-3 fails to reflect the effects of MUFAs [82]. Therefore, additional indices capable of accounting for the functional effects of fatty acids are used. The indexes IA, IT, HH, and FLQ were calculated in the lipid fraction. IA, IT, and HH are associated with the potential platelet aggregation and subsequent adverse risks at the cardiovascular system [85]. Lower IA and IT values are desirable to prevent cardiovascular disorder [37]. In the selected species, the IA index varied from 0.58 for deep water rose shrimp to 0.92 for red mullet and the IT values ranged from 0.27 for European pilchard to 0.60 for red mullet (Table 3), thus remaining within the range of the expected values [27]. On the contrary, a higher value of HH is more beneficial for human health [86]. The measured HH index ranged from 1.27 (red mullet) to 3.37 (European pilchard), the latest exceeding values reported for marine fish (0.87–2.46) [27,87]. The FLQ index is considered more suitable for fish or marine products due to their higher proportions of EPA and DHA [83]. The obtained FLQ values fall in the range of variability measured for different marine species [83] (and references therein). The highest values were registered in European pilchard (33.8) and European hake (31.2) (Table 3), confirming the well-known value of these species relating to their higher n-3 PUFA content [49,51,88].

## 4. Conclusions

This study represents the first approach to characterise the nutrient parameters of most widely commercialised fish species in Sicily (Italy, Mediterranean Sea). The variability of fatty acid concentrations, both at the intra- and inter-specific levels, reflect the different biological characteristics of the analysed species and, presumably, the different conditions of their feeding areas. Although this study presents some limitations, such as the absence of minerals and vitamins analysis, our results demonstrate the high nutritional values of the selected seafood and especially highlight the healthy fatty acid nutrient profile of the “blue fish” species such as European pilchard, which shows the highest total protein amount, the highest n3-PUFA content, and the best nutritional quality indices. This study encourages the consumption of low-cost marine target species of local, artisanal, and sustainable fisheries which are characteristic of the study area, highlighting the opportunity for consumers to take advantage of high-nutrient resources with affordable commercial value.

## Figures and Tables

**Table 1:** Biometrics data, number of individuals per site, and habitat of the species.

*Scientific Name*	Individuals per Site						Length	Weight	Habitat
(Common Name)	PT Porticello	SA S. Agata	CT Catania	PZ Pozzallo	SC Sciacca	MV Mazara del Vallo	(cm)	(g)	
*Sardina pilchardus*	46	50	50	55	59	/	15.3 ± 1.3	26.7 ± 7.9	pelagic
(European pilchard)									
*Merluccius merluccius*	11	3	9	26	17	14	23.5 ± 4.8	123.5 ± 128.1	demersal
(European hake)									
*Mullus surmuletus*	15	8	18	/	/	16	17.9 ± 2.1	73.1 ± 26.1	demersal
(Surmullet)									
*Mullus barbatus*	/	15	/	15	34	/	16.6 ± 1.9	53.8 ± 20	demersal
(Red mullet)									
*Parapenaeus longirostris*	126	88	130	104	158	111	3.8 ± 0.7 *	5.8 ± 2.6	demersal
(Deep water rose shrimp)									

(*) total length from the tip of the rostrum to the posterior edge of the carapace.

**Table 2:** Total protein content, total EAAs, and individual EAAs (% w.w.) of the selected species and relative sampling area.

				Amino Acid Fractions						
Species	Area	Total Protein	EAA	Val	Leu	ILe	Thr	Phen	Lys	Try
European pilchard	PT	24.6	11.2	1.8	3.3	1.6	1.6	0.3	1.0	1.6
	SA	25.4	13.6	2.1	3.1	1.8	1.7	0.4	2.7	1.8
	CT	23.9	12.5	2.3	3.1	1.9	1.7	0.4	1.4	1.6
	PZ	25.8	13.9	2.3	3.4	1.9	1.9	0.4	1.6	2.3
	SC	24.1	12.4	1.3	2.7	1.2	1.2	0.7	2.4	2.8
Mean ± Dev.St		24.8 ± 0.8	12.72 ± 1.06	1.94 ± 0.4	3.15 ± 0.26	1.69 ± 0.29	1.63 ± 0.27	0.45 ± 0.16	1.84 ± 0.71	2.02 ± 0.5
European hake	PT	18.4	9.6	1.4	2.3	1.2	1.0	0.3	1.9	1.5
	SA	19.6	6.4	1.1	1.5	0.9	0.8	0.3	1.1	0.7
	CT	17.3	9.0	1.4	2.6	1.2	1.0	0.3	1.3	1.2
	PZ	20.3	10.3	1.2	2.1	1.2	1.1	0.5	2.9	1.3
	SC	20.8	11.9	1.5	2.2	1.3	1.1	0.4	2.5	2.9
	MV	23.1	12.3	2.0	3.7	1.8	1.7	0.5	1.1	1.5
Mean ± Dev.St		19.9 ± 2	9.91 ± 2.15	1.42 ± 0.3	2.39 ± 0.75	1.27 ± 0.28	1.12 ± 0.3	0.4 ± 0.11	1.8 ± 0.77	1.51 ± 0.75
Surmullet	PT	24.0	12.0	2.0	3.4	1.8	1.6	0.4	1.0	1.8
	SA	23.1	12.5	1.5	2.1	1.7	1.3	0.8	3.2	2.0
	CT	24.3	12.8	2.0	3.0	1.7	1.6	0.4	2.5	1.7
	MV	19.8	10.1	1.7	2.9	1.5	1.3	0.4	1.0	1.4
Mean ± Dev.St		22.8 ± 2.1	9.93 ± 1.21	1.52 ± 0.26	2.42 ± 0.51	1.39 ± 0.12	1.21 ± 0.2	0.41 ± 0.18	1.69 ± 1.09	1.53 ± 0.25
Red mullet	SA	23.3	10.9	1.6	2.6	1.3	1.4	0.3	1.4	2.4
	PZ	23.4	12.9	1.9	3.3	1.6	1.3	0.4	2.3	2.0
	SC	23.0	10.3	1.7	2.6	1.5	1.3	0.3	1.0	1.7
Mean ± Dev.St		23.2 ± 0.2	11.34 ± 1.35	1.71 ± 0.14	2.82 ± 0.43	1.48 ± 0.13	1.35 ± 0.01	0.36 ± 0.07	1.57 ± 0.66	2.04 ± 0.32
Deep water rose shrimp	PT	20.7	10.8	1.5	2.6	1.4	1.1	0.3	2.1	1.7
	SA	21.3	8.2	1.5	2.2	1.4	1.6	0.3	1.6	1.3
	CT	20.3	9.2	1.4	1.9	1.3	1.7	0.3	1.2	1.3
	PZ	18.3	9.3	1.2	2.0	1.1	1.4	0.4	1.6	1.6
	SC	18.0	8.3	1.2	1.7	1.2	1.5	0.2	1.1	1.2
	MV	22.2	10.5	1.5	2.5	1.5	2.1	0.5	1.4	1.1
Mean ± Dev.St		20.1 ± 1.7	9.37 ± 1.06	1.4 ± 0.15	2.16 ± 0.34	1.31 ± 0.13	1.56 ± 0.31	0.33 ± 0.09	1.49 ± 0.37	1.35 ± 0.22

**Table 3:** Mean ± standard deviation of total fatty acid content (% w.w.) and fatty acid composition (% of TFA) in the selected species from different sampling areas.

Specie	Area	Total FA	ΣSFA	ΣMUFA	ΣPUFA	C20:5n3 (EPA)	C22:6n3 (DHA)
European pilchard	CT	8.96 ± 1.32	26.8 ± 4.16	19.8 ± 2.89	51.9 ± 6.05	15.2 ± 2.62	29.9 ± 2.68
	MV	4.69 ± 1.13	38.6 ± 14.4	19.8 ± 3.75	40.3 ± 11.0	9.18 ± 2.58	25.9 ± 7.22
	PT	5.87 ± 1.03	38.1 ± 9.67	20.5 ± 3.00	40.4 ± 6.00	11.8 ± 1.33	22.8 ± 3.50
	PZ	2.63 ± 1.82	51.3 ± 3.99	16.0 ± 4.39	32.5 ± 3.61	5.55 ± 1.26	22.1 ± 3.98
	SA	3.17 ± 0.74	50.4 ± 3.52	17.9 ± 1.32	31.2 ± 3.73	6.44 ± 0.71	20.0 ± 2.80
European hake	CT	1.07 ± 0.45	45.4 ± 3.21	16.0 ± 2.67	36.4 ± 1.73	4.61 ± 0.46	25.8 ± 1.05
	MV	1.64 ± 0.44	43.3 ± 1.50	14.6 ± 0.85	38.6 ± 2.52	6.19 ± 0.41	27.3 ± 2.05
	PT	0.97 ± 0.24	53.1 ± 2.96	16.1 ± 2.71	24.0 ± 2.55	3.71 ± 0.55	16.3 ± 1.78
	PZ	1.69 ± 1.13	42.5 ± 5.57	12.1 ± 2.18	44.0 ± 4.56	5.29 ± 0.63	33.2 ± 4.21
	SA	1.79 ± 0.31	38.1 ± 2.47	10.9 ± 0.85	50.7 ± 1.94	4.74 ± 0.76	41.0 ± 0.54
	SC	1.97 ± 0.46	44.4 ± 1.91	15.4 ± 3.32	37.7 ± 2.96	5.36 ± 0.51	27.0 ± 2.72
Surmullet	CT	2.86 ± 0.58	47.8 ± 1.94	34.4 ± 2.47	16.1 ± 2.63	4.60 ± 0.39	5.29 ± 2.45
	MV	1.51 ± 0.59	45.6 ± 3.18	10.1 ± 2.23	44.3 ± 3.72	8.45 ± 1.00	27.1 ± 3.86
	PT	2.35 ± 0.33	50.4 ± 1.73	29.4 ± 1.86	17.0 ± 1.00	4.08 ± 0.32	5.47 ± 0.37
	SA	2.10 ± 0.36	50.7 ± 3.72	23.1 ± 3.87	23.3 ± 3.19	6.01 ± 1.95	10.2 ± 1.70
Red mullet	PZ	2.19 ± 1.37	55.9 ± 8.68	22.1 ± 6.62	20.2 ± 8.66	5.52 ± 2.44	7.79 ± 4.38
	SA	2.75 ± 0.73	52.7 ± 2.20	22.2 ± 1.38	23.2 ± 2.53	6.88 ± 1.47	8.50 ± 1.87
	SC	2.10 ± 1.19	52.5 ± 2.98	25.6 ± 4.90	19.4 ± 2.15	5.24 ± 0.48	6.48 ± 1.38
Deep water rose shrimp	CT	2.03 ± 0.50	44.3 ± 3.61	23.1 ± 3.15	30.9 ± 2.47	10.7 ± 1.17	12.7 ± 1.79
	MV	1.38 ± 0.37	49.4 ± 8.11	20.4 ± 0.95	28.1 ± 6.97	10.6 ± 3.26	11.7 ± 3.98
	PT	2.37 ± 0.39	36.8 ± 0.51	22.8 ± 1.26	39.9 ± 1.34	14.0 ± 0.83	16.0 ± 1.08
	PZ	1.75 ± 0.29	45.1 ± 1.38	21.9 ± 2.22	30.5 ± 2.86	10.4 ± 0.39	10.7 ± 1.32
	SA	1.78 ± 0.34	40.5 ± 1.68	21.6 ± 2.61	37.1 ± 1.19	12.3 ± 0.27	13.9 ± 0.76
	SC	2.09 ± 0.05	42.8 ± 1.29	20.3 ± 1.21	36.8 ± 0.37	11.1 ± 1.23	18.3 ± 1.98

**Table 4:** Nutritional quality indexes of the lipid fraction of the selected species.

Nutritional Indexes	European Pilchard	European Hake	Surmullet	Red Mullet	Deep Water Rose Shrimp
n6/n3	0.08	0.06	0.13	0.19	0.09
PUFA/SFA	1.21	0.86	0.59	0.41	0.84
FLQ	33.83	31.20	19.85	13.48	25.52
IA	0.78	0.72	0.79	0.92	0.58
IT	0.27	0.29	0.47	0.60	0.30
HH	3.37	1.96	1.54	1.27	2.12

## Data Availability

The datasets used and analysed during the current study are available from the corresponding author.

## Supplementary Materials

The following supporting information can be downloaded at https://www.mdpi.com/article/10.3390/ani14152158/s1. Table S1: Mean ± standard deviation of total fatty acids content (% w.w.) and fatty acid composition (% of TFA) in European pilchard and European hake from different areas. Table S2: Mean ± standard deviation of total fatty acids content (% w.w.) and fatty acid composition (% of TFA) in surmullet, red mullet and deep water rose shrimp from different areas.
