# Supplementary material for: Protein, Essential Amino Acid, and Fatty Acid Composition of Five Target Fishery Species of Central Mediterranean Sea
Source: Animals (Basel). 2024 Jul 24;14(15):2158. doi: 10.3390/ani14152158 (PMC11310956; doi:10.3390/ani14152158)
Supplement: Supplementary file 1 [file animals-14-02158-s001.zip › animals-3070983-supplementary.pdf]

Table S1

	S. pilchardus (European pilchard)					*M. merluccius* (European hake)					
	**CT**	**MV**	**PT**	**PZ**	**SA**	**CT**	**MV**	**PT**	**PZ**	**SA**	**SC**
**Lipidi Totale % (w.w.)**	8.96 ± 1.32	4.69 ± 1.13	5.87 ± 1.03	2.63 ± 1.82	3.17 ± 0.74	1.07 ± 0.45	1.64 ± 0.44	0.97 ± 0.24	1.69 ± 1.13	1.79 ± 0.31	1.97 ± 0.46
**C14:0**	8.25 ± 0.48	6.31 ± 0.90	6.90 ± 1.05	5.84 ± 0.42	5.45 ± 0.51	4.29 ± 2.43	3.44 ± 0.56	4.92 ± 1.15	4.30 ± 1.79	2.65 ± 0.26	3.65 ± 0.33
**C15:0**	1.68 ± 0.14	1.91 ± 0.46	1.78 ± 0.12	2.67 ± 1.08	2.17 ± 0.37	2.44 ± 2.05	2.04 ± 1.18	3.41 ± 1.69	2.63 ± 2.19	2.51 ± 0.62	2.53 ± 0.47
**C16:0**	4.87 ± 5.64	15.9 ± 14.9	14.8 ± 12.6	25.5 ± 0.71	27.3 ± 1.22	20.9± 1.70	22.2 ± 0.36	19.2 ± 2.07	18.4± 0.81	18.4 ± 0.63	21.0 ± 1.43
**C17:0**	1.68 ± 0.17	2.06 ± 0.52	1.97 ± 0.12	2.89 ± 1.14	2.49 ± 0.36	3.51 ± 2.05	3.20 ± 0.76	4.87 ± 1.43	4.02 ± 1.88	2.71 ± 0.63	2.94 ± 0.50
**C18:0**	6.69 ± 0.81	8.06 ± 1.52	7.59 ± 0.70	8.97 ± 1.52	8.13 ± 0.57	10.9 ± 2.01	9.37 ± 1.43	12.6 ± 0.87	11.0 ± 1.41	8.29 ± 0.20	10.5 ± 1.17
**C20:0**	0.99 ± 0.09	0.99 ± 0.56	0.65 ± 0.75	2.40 ± 1.14	1.13 ± 1.32	0.96 ± 1.51	1.35 ± 1.26	4.46 ± 1.69	1.34 ± 1.33	2.27 ± 0.43	1.95 ± 1.15
**C21:0**	0.39 ± 0.27	1.09 ± 0.61	0.60 ± 0.44	0.57 ± 0.70	0.92 ± 0.65	n.d	n.d	n.d	n.d	n.d	n.d
**C22:0**	0.72 ± 0.08	0.19 ± 0.42	0.47 ± 0.55	0.00 ± 0.00	0.00 ± 0.00	n.d	n.d	0.52 ± 1.37	n.d	n.d	n.d
**C23:0**	0.51 ± 0.08	0.45 ± 0.42	0.79 ± 0.18	0.21 ± 0.42	0.53 ± 0.61	n.d	n.d	n.d	n.d	n.d	n.d
**C24:0**	1.03 ± 1.22	1.58 ± 0.92	2.55 ± 0.28	2.30 ± 0.20	2.30 ± 0.15	2.38 ± 0.54	1.64 ± 0.34	3.14 ± 0.53	0.81 ± 0.77	1.29 ± 0.29	1.83 ± 0.11
**C15:1**	0.13 ± 0.03	0.10 ± 0.07	0.09 ± 0.07	n.d	n.d	n.d	n.d	n.d	n.d	n.d	n.d
**C16:1**	6.25 ± 4.21	3.90 ± 0.72	6.67 ± 0.91	2.57 ± 0.86	3.01 ± 0.30	2.18 ± 0.48	1.11 ± 0.13	1.14 ± 0.38	1.53 ± 0.20	1.36 ± 0.10	1.95 ± 0.58
**C18:1n9t**	0.44 ± 0.06	0.36 ± 0.20	0.37 ± 0.25	0.26 ± 0.31	0.64 ± 0.13	n.d	n.d	n.d	n.d	n.d	n.d
**C18:1n9c**	10.2 ± 1.49	12.8 ± 1.88	10.4 ± 1.36	10.2 ± 2.63	11.2 ± 1.27	11.2 ± 1.33	11.7 ± 0.74	10.9 ± 2.49	8.96 ± 0.65	8.39 ± 0.84	10.8 ± 0.97
**C20:1n9c**	1.46 ± 0.16	1.61 ± 0.93	1.65 ± 0.17	2.26 ± 0.44	1.95 ± 0.20	1.59 ± 0.86	1.80 ± 0.17	1.98 ± 0.30	0.69 ± 0.65	1.15 ± 0.09	1.16 ± 0.70
**C22:1n9**	0.81 ± 0.02	0.55 ± 0.31	0.94 ± 0.08	0.70 ± 0.50	1.08 ± 0.12	n.d	n.d	n.d	n.d	n.d	n.d
**C24:1n9**	0.59 ± 0.08	0.53 ± 0.49	0.40 ± 0.46	n.d	n.d	1.04 ± 1.64	n.d	2.07 ± 2.62	0.93 ± 1.30	n.d	1.44 ± 1.34
**C18:2n6c**	2.29 ± 0.27	2.26 ± 0.32	2.28 ± 0.31	1.97 ± 0.05	2.11 ± 0.08	2.07 ± 0.20	1.80 ± 0.08	1.94 ± 0.15	1.90 ± 0.12	1.25 ± 0.05	2.07 ± 0.18
**C20:2**	0.57 ± 0.10	0.09 ± 0.20	n.d	0.12 ± 0.24	0.12 ± 0.23	0.21 ± 0.24	0.34 ± 0.19	0.27 ± 0.25	0.11 ± 0.17	0.36 ± 0.01	0.33 ± 0.21
**C22:2**	0.09 ± 0.01	n.d	0.07 ± 0.05	n.d	n.d	n.d	n.d	n.d	n.d	n.d	n.d
**C18:3n6c**	0.26 ± 0.03	0.21 ± 0.12	0.27 ± 0.02	0.14 ± 0.17	0.14 ± 0.16	n.d	n.d	n.d	n.d	n.d	n.d
**C18:3n3c**	0.94 ± 0.11	1.09 ± 0.17	0.90 ± 0.13	0.89 ± 0.07	0.92 ± 0.07	0.48 ± 0.54	0.65 ± 0.37	0.42 ± 0.43	0.29 ± 0.27	0.58 ± 0.09	0.59 ± 0.39
**C20:3n6c**	0.38 ± 0.02	0.24 ± 0.14	0.39 ± 0.05	0.17 ± 0.19	0.14 ± 0.16	n.d	n.d	n.d	n.d	n.d	n.d
**C20:3n3c**	2.19 ± 0.28	1.16 ± 0.68	1.55 ± 1.04	1.48 ± 0.11	1.13 ± 0.10	3.16 ± 0.61	2.34 ± 0.33	2.38 ± 0.48	3.19 ± 0.69	2.86 ± 0.78	2.26 ± 0.48
**C20:4n6c**	0.21 ± 0.03	0.20 ± 0.11	0.24 ± 0.01	0.06 ± 0.12	0.15 ± 0.17	n.d	n.d	n.d	n.d	n.d	n.d
**C20:5n3 (EPA)**	15.2 ± 2.62	9.18 ± 2.58	11.8 ± 1.33	5.55 ± 1.26	6.44 ± 0.71	4.61 ± 0.46	6.19 ± 0.41	3.71 ± 0.55	5.29 ± 0.63	4.74 ± 0.76	5.36 ± 0.51
**C22:6n3 (DHA)**	29.9 ± 2.68	25.9 ± 7.22	22.8 ± 3.50	22.1 ± 3.98	20.0 ± 2.80	25.8 ± 1.05	27.3 ± 2.05	16.3 ± 1.78	33.2 ± 4.21	41.0 ± 0.54	27.0 ± 2.72

**ΣSFA**	26.8 ± 4.16	38.6 ± 14.4	38.1 ± 9.67	51.3 ± 3.99	50.4 ± 3.52	45.4 ± 3.21	43.3 ± 1.50	53.1 ± 2.96	42.5 ± 5.57	38.1 ± 2.47	44.4 ± 1.91
**ΣMUFA**	19.8 ± 2.89	19.8 ± 3.75	20.5 ± 3.00	16.0 ± 4.39	17.9 ± 1.32	16.0 ± 2.67	14.6 ± 0.85	16.1 ± 2.71	12.1 ± 2.18	10.9 ± 0.85	15.4 ± 3.32
**ΣPUFA**	51.9 ± 6.05	40.3 ± 11.0	40.4 ± 6.00	32.5 ± 3.61	31.2 ± 3.73	36.4 ± 1.73	38.6 ± 2.52	24.0 ± 2.55	44.0 ± 4.56	50.7 ± 1.94	37.7 ± 2.96

Table S2

	M. surmuletus (surmullet)				*M. barbatus* (red mullet)			*P. longirostris* (Deep water rose shrimp)					
	CT	MV	PT	SA	PZ	SA	SC	CT	MV	PT	PZ	SA	SC
**Lipidi Totale % (w.w.)**	2.86 ± 0.58	1.51 ± 0.59	2.35 ± 0.33	2.10 ± 0.36	2.19 ± 1.37	2.75 ± 0.73	2.10 ± 1.19	2.03 ± 0.50	1.38 ± 0.37	2.37 ± 0.39	1.75 ± 0.29	1.78 ± 0.34	2.09 ± 0.05
**C14:0**	3.35 ± 0.33	4.01 ± 0.61	3.62 ± 0.34	4.36 ± 0.37	4.40 ± 1.33	4.41 ± 0.27	3.88 ± 0.74	2.64 ± 0.63	4.11 ± 1.85	2.12 ± 0.21	2.78 ± 0.47	2.71 ± 0.30	2.21 ± 0.07
**C15:0**	2.29 ± 0.36	3.07 ± 1.39	2.66 ± 0.38	2.94 ± 0.34	3.92 ± 1.85	3.02 ± 0.93	3.59 ± 0.79	2.76 ± 0.80	4.07 ± 1.97	2.27 ± 0.19	2.97 ± 0.43	2.91 ± 0.49	2.59 ± 0.15
**C16:0**	27.5 ± 1.23	22.8 ± 2.63	26.6 ± 0.45	22.5 ± 3.79	23.8 ± 4.87	24.7 ± 0.54	22.9 ± 1.48	22.5 ± 1.22	22.3 ± 1.07	18.4 ± 1.03	19.8 ± 0.45	19.8 ± 0.07	22.7± 2.06
**C17:0**	2.93 ± 0.39	4.14 ± 0.74	3.52 ± 0.35	3.62 ± 0.64	4.82 ± 1.98	3.87 ± 0.90	4.37 ± 1.00	3.66 ± 1.13	5.01 ± 1.93	2.92 ± 0.17	3.76 ± 0.41	3.72 ± 0.71	3.60 ± 0.25
**C18:0**	8.55 ± 0.70	10.2 ± 0.82	9.88 ± 0.68	12.0 ± 1.98	12.72 ± 2.15	12.7 ± 0.69	11.8 ± 1.55	9.49± 1.78	11.3 ± 1.76	8.05 ± 0.10	10.2 ± 0.34	9.27 ± 0.99	10.0 ± 0.67
**C20:0**	2.10 ± 0.38	0.61 ± 1.17	2.71 ± 0.47	2.92 ± 0.47	2.88 ± 2.83	2.60 ± 0.64	2.81 ± 0.93	1.51 ± 0.85	n.d	0.29 ± 0.65	1.72 ± 1.17	0.97 ± 1.12	0.00.± 0.00
**C21:0**	n.d	n.d	n.d	n.d	0.84 ± 1.12	0.55 ± 0.80	1.62 ± 1.08	n.d	1.94 ± 2.24	n.d	n.d	n.d	n.d
**C22:0**	n.d	0.22 ± 0.61	n.d	0.74 ± 1.49	1.58 ± 3.17	n.d	0.26 ± 0.58	1.12 ± 1.02	n.d	0.72 ± 1.01	2.53 ± 0.36	n.d	1.02 ± 1.18
**C23:0**	0.45 ± 0.70	n.d	0.39 ± 0.88	n.d	n.d	n.d	0.20 ± 0.45	n.d	n.d	n.d	n.d	n.d	n.d
**C24:0**	0.65 ± 0.56	0.49 ± 0.69	1.02 ± 0.60	1.61 ± 0.28	0.91 ± 1.42	0.87 ± 0.52	1.07 ± 0.35	0.66 ± 0.38	0.72 ± 1.45	1.56 ± 0.63	1.36 ± 0.12	1.09 ± 0.23	0.64 ± 0.74
**C15:1**	0.23 ± 0.14	0.04 ± 0.12	0.33 ± 0.13	0.59 ± 0.39	0.31 ± 0.37	0.37 ± 0.36	0.59 ± 0.14	0.45 ± 0.05	0.31 ± 0.21	0.19 ± 0.18	0.39 ± 0.06	0.47 ± 0.02	0.33 ± 0.01
**C16:1**	5.21 ± 0.44	1.97 ± 0.68	4.69 ± 0.22	4.14 ± 1.95	4.96 ± 2.46	5.56 ± 0.75	5.53 ± 1.45	5.71 ± 1.77	3.43 ± 1.11	3.45 ± 1.09	4.11 ± 0.56	3.98 ± 1.66	2.18 ± 0.18
**C18:1n9t**	0.50 ± 0.41	0.09 ± 0.27	0.99 ± 0.13	1.00 ± 0.70	0.23 ± 0.45	0.48 ± 0.67	1.17 ± 0.34	0.79 ± 0.44	0.94 ± 1.23	0.72 ± 0.41	1.14 ± 0.16	1.17 ± 0.11	0.44 ± 0.51
**C18:1n9c**	25.0 ± 2.54	6.91 ± 1.56	20.5 ± 1.38	14.5 ± 2.92	14.8 ± 4.80	13.9 ± 0.58	15.3 ± 3.64	14.9 ± 1.43	14.4 ± 1.90	16.6 ± 1.48	14.1 ± 0.96	14.3 ± 1.22	16.5 ± 0.46
**C20:1n9c**	2.35 ± 0.16	0.98 ± 0.83	2.27 ± 0.19	2.02 ± 0.50	1.69 ± 1.35	1.80 ± 0.35	2.23 ± 0.18	1.32 ± 0.36	1.27 ± 1.56	1.79 ± 0.14	1.64 ± 0.13	1.64 ± 0.15	0.88 ± 1.02
**C22:1n9**	0.84 ± 0.22	0.09 ± 0.26	0.57 ± 0.53	0.82 ± 0.58	0.15 ± 0.29	0.11 ± 0.24	0.76 ± 0.46	n.d	n.d	n.d	n.d	n.d	n.d
**C24:1n9**	0.24 ± 0.59	n.d	n.d	n.d	n.d	n.d	n.d	n.d	n.d	n.d	0.54 ± 1.08	n.d	n.d
**C18:2n6c**	1.98 ± 0.19	1.72 ± 0.16	2.07 ± 0.19	2.14 ± 0.22	1.75 ± 0.17	2.02 ± 0.14	1.69 ± 0.21	1.63 ± 0.45	2.11 ± 0.10	1.52 ± 0.12	1.64 ± 0.14	2.09 ± 0.22	2.06 ± 0.17
**C20:2**	0.57 ± 0.29	0.24 ± 0.33	0.68 ± 0.38	0.84 ± 0.18	0.40 ± 0.48	0.62 ± 0.58	0.79 ± 0.11	0.88 ± 0.53	0.45 ± 0.90	1.43 ± 0.21	1.45 ± 0.25	1.72 ± 0.26	0.90 ± 1.04
**C22:2**	0.08 ± 0.19	n.d	n.d	n.d	n.d	n.d	n.d	n.d	n.d	n.d	n.d	n.d	n.d
**C18:3n6c**	0.27 ± 0.22	0.05 ± 0.15	0.34 ± 0.32	0.00 ± 0.00	0.92 ± 0.39	0.62 ± 0.48	0.91 ± 0.16	0.40 ± 0.23	0.16 ± 0.31	0.55 ± 0.08	0.66 ± 0.07	0.27 ± 0.31	0.59 ± 0.03
**C18:3n3c**	0.72 ± 0.08	0.06 ± 0.17	0.8 ± 0.15	0.60 ± 0.40	0.10 ± 0.21	0.26 ± 0.36	0.17 ± 0.25	0.45 ± 0.25	0.63 ± 0.42	0.46 ± 0.26	0.15 ± 0.31	0.30 ± 0.35	0.70 ± 0.06
**C20:3n6c**	0.18 ± 0.20	0.00 ± 0.00	0.41 ± 0.24	0.26 ± 0.31	0.44 ± 0.31	0.10 ± 0.22	0.46 ± 0.26	0.08 ± 0.18	0.14 ± 0.27	n.d	n.d	0.12 ± 0.24	0.08 ± 0.16
**C20:3n3c**	2.38 ± 0.41	6.58 ± 3.17	3.19 ± 0.31	3.24 ± 0.59	3.22 ± 2.89	4.14 ± 0.49	3.66 ± 0.58	3.44 ± 1.98	2.16 ± 2.60	5.62 ± 0.43	5.01 ± 0.43	5.98 ± 0.64	2.25 ± 2.61
**C20:4n6c**	n.d	0.04 ± 0.12	n.d	n.d	0.08 ± 0.16	0.08 ± 0.18	n.d	0.58 ± 0.20	0.20 ± 0.39	0.33 ± 0.30	0.46 ± 0.31	0.43 ± 0.29	0.70 ± 0.07
**C20:5n3 (EPA)**	4.60 ± 0.39	8.45 ± 1.00	4.08 ± 0.32	6.01 ± 1.95	5.52 ± 2.44	6.88 ± 1.47	5.24 ± 0.48	10.7 ± 1.17	10.6 ± 3.26	14.0 ± 0.83	10.4 ± 0.39	12.3 ± 0.27	11.1 ± 1.23
**C22:6n3 (DHA)**	5.29 ± 2.45	27.1 ± 3.86	5.47 ± 0.37	10.2 ± 1.70	7.79 ± 4.38	8.50 ± 1.87	6.48 ± 1.38	12.7 ± 1.79	11.7 ± 3.98	16.0 ± 1.08	10.7 ± 1.32	13.9 ± 0.76	18.3 ± 1.98

**ΣSFA**	47.8 ± 1.94	45.6 ± 3.18	50.4 ± 1.73	50.7 ± 3.72	55.9 ± 8.68	52.7 ± 2.20	52.5 ± 2.98	44.3 ± 3.61	49.4 ± 8.11	36.8 ± 0.51	45.1 ± 1.38	40.5 ± 1.68	42.8 ± 1.29
**ΣMUFA**	34.4 ± 2.47	10.1 ± 2.23	29.4 ± 1.86	23.1 ± 3.87	22.1 ± 6.62	22.2 ± 1.38	25.6 ± 4.90	23.1 ± 3.15	20.4 ± 0.95	22.8 ± 1.26	21.9 ± 2.22	21.6 ± 2.61	20.3 ± 1.21
**ΣPUFA**	16.1 ± 2.63	44.3 ± 3.72	17.0 ± 1.00	23.3 ± 3.19	20.2 ± 8.66	23.2 ± 2.53	19.4 ± 2.15	30.9 ± 2.47	28.1 ± 6.97	39.9 ± 1.34	30.5 ± 2.86	37.1 ± 1.19	36.8 ± 0.37
